# Experimental Investigation on Post-Fire Mechanical Properties of Glass Fiber-Reinforced Polymer Rebars

**DOI:** 10.3390/polym15132925

**Published:** 2023-07-01

**Authors:** Chanachai Thongchom, Lili Hu, Penpichcha Khongpermgoson Sanit-in, Denise-Penelope N. Kontoni, Nitipong Praphaphankul, Koravith Tiprak, Suphanut Kongwat

**Affiliations:** 1 Department of Civil Engineering, Faculty of Engineering, Thammasat School of Engineering, Thammasat University, Pathumthani 12120, Thailand; tchanach@engr.tu.ac.th; 2 State Key Laboratory of Ocean Engineering, School of Naval Architecture, Ocean and Civil Engineering, Shanghai Jiao Tong University, Shanghai 200240, China; 3 Department of Civil Engineering, Faculty of Engineering, Kasetsart University Kamphaeng Saen Campus, Nakhon Pathom 73140, Thailand; fengppcs@ku.ac.th; 4 Department of Civil Engineering, School of Engineering, University of the Peloponnese, GR-26334 Patras, Greece; kontoni@uop.gr; 5 School of Science and Technology, Hellenic Open University, GR-26335 Patras, Greece; 6 Department of Civil and Environmental Engineering, Tokyo Institute of Technology, 2-12-1-M1-23 Ookayama, Meguro-ku, Tokyo 152-8552, Japan; praphaphankul.n.aa@m.titech.ac.jp (N.P.); tiprak.k.aa@m.titech.ac.jp (K.T.); 7 Department of Mechanical Engineering, Faculty of Engineering, King Mongkut’s University of Technology Thonburi, Bangkok 10140, Thailand; suphanut.kon@kmutt.ac.th; 8 Future Automotive Structure Research Group (FASt), Mobility and Vehicle Technology Research Center, King Mongkut’s University of Technology Thonburi, Bangkok 10140, Thailand

**Keywords:** post-fire strength, glass fiber-reinforced polymer (GFRP) bar, tensile strength, tensile behavior, temperature

## Abstract

Glass fiber-reinforced polymer (GFRP) rebars are commonly used as an alternative to conventional steel reinforcement in a variety of structural applications due to their superior low cost, strength-to-weight ratio, and durability. However, their mechanical properties after exposure to elevated temperatures, particularly in fire-prone environments, remain a significant concern. To address this concern, the present study focuses on investigating the residual tensile behavior, specifically the tensile strength and elastic modulus, of GFRP rebars exposed to high temperatures that are realistically encountered during fire incidents. The temperature range considered in this analysis spans from 100 °C to 400 °C, with a heating rate of 20 °C/min. The fire duration of 1 h is used. This comprehensive analysis is essential for enhancing our understanding of the performance and applicability of GFRP rebars in fire-prone environments. Based on their actual application in the construction industry, five specimens of three different rebar sizes (16, 20, and 25 mm) were examined for the effect of rebar size on tensile behavior after fire exposure. In addition, the effects were investigated of air- and water-cooling methods on residual tensile behavior. The nominal tensile strength, elastic modulus, and ultimate strain of GFRP rebars at ambient temperature are 930 MPa, 50.2 GPa and 1.85%, respectively. The test results indicated that as the temperature increased to 400 °C, the ultimate tensile strength of the GFRP bars decreased by up to 55%, while the ultimate strain increased by up to 44%, regardless of the cooling method. In addition, when rebars of sizes 16–25 mm were subjected to a 400 °C fire treatment, the smaller the rebar, the greater the percentage of ultimate tensile and strain reduction. These findings hold great significance for the utilization of GFRP bars within the construction industry. This study offers valuable insights into the design of fire-resilient structures, emphasizing the importance of considering rebar size and cooling methods due to their impact on the post-fire tensile strength and strain of GFRP rebars.

## 1. Introduction

Reinforcing steel in concrete is a vital material used to enhance structural strength. In addition to its usability, it is crucial to continuously assess its deterioration to evaluate the structure’s usability and lifespan. However, the deterioration of reinforcing steel in concrete is influenced by various factors, including its mechanical and physical properties, as well as exposure to environmental conditions and different types of disasters, such as fire resistance and corrosion caused by environments with high concentrations of acids or solutions.

Corrosion of the reinforcements in reinforced concrete (RC) has long been recognized as a major factor in the deterioration of the performance, especially in environments where chlorides are present, such as those found near the ocean or where deicing salts are used [1]. Consequently, structural damage can appear regularly throughout the structure’s service life. This damage can manifest as cracking and spalling of the cover concrete, ultimately affecting the RC element’s load-bearing and shear capacities [2]. Fiber-reinforced polymer (FRP) corrosion-resistant rebars have been considered to address these issues [3]. Nowadays, FRP are employed in a diverse range of civil engineering applications, including as rebars, plates, sheets and pultruded profiles. The use of pultruded profiles is confronted with challenges pertaining to shape distortions over time [4,5]. Consequently, the rebar form, owing to its inherent stability and dependability, has attracted substantial interest and is extensively deployed in infrastructures such as bridges, buildings, and industrial structures. Furthermore, glass fiber-reinforced plastic (GFRP) rebars have been promoted for use in civil engineering due to their low cost, high strength-to-weight and stiffness-to-weight ratios, and corrosion resistance [6]. Despite the fact that the usage of GFRP rebars has been codified [7,8,9] and is extensively recognized within the industry [10,11,12], these materials exhibit an inclination for degradation under severe environmental conditions [13]. Research and real-world applications have demonstrated this material’s effectiveness in harsh environments involving seawater and sea sand concrete, seismicity, ultraviolet radiation, and water vapor condensation [14,15,16]. Nevertheless, concrete reinforced with GFRP rebars exposed to high temperatures or fire requires additional research and analysis to accurately assess the residual capacity of the structure and to understand its behavior in greater depth.

Some research indicates that the mechanical properties of GFRP rebars degrade at high temperatures [17,18,19,20,21,22,23]. For example, the tensile strength and elastic modulus of GFRP rebars tested at 325 °C decreased by 55% and 30%, respectively [19]. At 350 °C, the initial tensile strength of both GFRP and carbon fiber-reinforced plastic (CFRP) rebars decreased by 45 and 35%, respectively [18]. Notably, the size effect of GFRP rebars is important when applied in real-world engineering. However, only limited studies have focused on the behavior of GFRP rebars with various diameters. For example, only 13 mm diameter rebars were mentioned in the study by Bisby et al. [17], 10 mm diameter rebars are mentioned in the study by Hamad et al. [19], and 9.5 and 12.7 mm diameter rebars are mentioned in the study by Wang et al. [18]. Thus, additional major evidence is required on the behavior of the various GFRP rebar sizes and cooling methods after high-temperature exposure.

In addition, the published literature on conventional steel-reinforced concrete has discussed how the procedure of cooling the temperature of the structure affects the structural performance after high-temperature exposure. For example, Lee et al. [24] found that the bond strength of specimens cooled with water was greater than that of specimens cooled naturally. The distractive effect of water cooling was greater than that of air cooling, particularly for longer heating durations [25]. The post-fire compressive strength, compressive strength, and modulus of elasticity of concrete reinforced with CFRP wraps were affected by the cooling technique, with water cooled specimens experiencing a greater reduction in strength than air-cooled specimens [26]. However, that study focused primarily on FRP sheet reinforcement and not rebar reinforcement.

To comprehensively clarify the mechanical properties of GFRP rebars under elevated temperature conditions—a prevalent scenario in structures susceptible to fire—and in consideration of the industry-standard rebar dimensions and cooling techniques, the current investigation examines the residual tensile behavior of GFRP rebars with diameters ranging from 16 to 25 mm. This examination employs both air-cooling and water-cooling methods. In addition, this research probes into the post-fire mechanical properties of GFRP rebars, with an emphasis on failure mode, the stress–strain relationship, tensile strength, and ultimate strain of GFRP rebars. The investigation was conducted using an ASTM D7205/D7205M-06 standard [27] tensile test with different GFRP rebar diameters, cooling methods, and elevated temperatures (100–400 °C). This study evaluated the tensile performance of GFRP rebars embedded in concrete sleeves subjected to high temperatures with varying rebar sizes and cooling techniques using an ASTM D7205/D7205M-06 standard tensile test [27]. The investigated temperature range provided the information necessary for analyses following various fire scenarios. In the evaluation of how various factors affected the tensile behavior of the GFRP rebars, normalization was applied to the tensile test results in addition to the experimental results to observe changes in the mechanical properties after a fire.

## 2. Experimental Program

This research investigated the post-fire mechanical properties of GFRP rebars, considering rebar size, maximum temperature exposure, and cooling technique. The experiment comprised 27 testing combinations, with a total of 135 specimens. These specimens consisted of three nominal diameters (16, 20, and 25 mm). Each rebar size was subjected to four different maximum temperatures (100, 200, 300, and 400 °C), while some specimens were maintained at ambient temperature (approximately 28 °C) as the control. After heating, the specimens were further divided into two groups to conduct the two different cooling methods of air or water cooling. Then, the specimens were left for 24 h to ensure that all of them reach a steady state at the ambient temperature before conducting the tensile tests.

### 2.1. Test Specimens

Rebar sizes of 16, 20, and 25 mm were used because they are the most commonly in the construction industry. In terms of the maximum temperature exposure, a literature review [28] reported that once GFRP rebars are exposed to maximum temperatures between 300 °C and 400 °C, their mechanical properties start to degrade. Thus, the mechanical properties after exposure to maximum temperatures of 100, 200, 300, and 400 °C were investigated in the current study and compared to an unexposed specimen. In addition, both air- and water-cooling methods were employed to simulate real-world scenarios involving post-fire reinforcement of GFRP rebars in structures. Specifically, the water-cooling technique represents situations where the structures have been subjected to fire and subsequently extinguished using water from a fire-fighting system. On the other hand, the air-cooling methods simulate portions of the structures that self-extinguished without the use of any external fire-fighting systems.

The 27 testing combinations, with 5 specimens each, are listed in Table 1. The first three combinations namely G16, G20, and G25 have rebar of sizes 16, 20, and 25 mm in diameter, respectively. Specimens in these three combinations were left at ambient temperature (approximately 25 °C), with no added heating or cooling processes performed. On the other hand, the remaining combinations were divided by considering not only the rebar size but also maximum temperature exposure and cooling approach. To clarify, different combinations were exposed to 4 different maximum temperatures (100, 200, 300, and 400 °C) and 2 different cooling techniques (air or water cooling). Therefore, the remaining combinations were composed of 3 × 4 × 2 = 24 combinations amounting to 24 × 5 = 120 specimens. For a clear understanding of the testing methods conducted in each combination, the testing combinations were named systematically. “G dd - ttt c” where “dd”, “ttt”, and “c” indicate the rebar nominal diameter (mm), maximum temperature exposure (°C), as well as whether water cooled (W) or air cooled (A), respectively. For example, “G20-200W” is the group of five replicates for rebars with 20 mm in nominal diameter that were heated up to 200 °C before conducting the tensile tests after 24 h of water cooling.

All the GFRP rebars were composed of continuous Advantex glass fibers inserted in a vinyl ester matrix using pultrusion. According to the ASTM D792 standard [29], the glass fraction was confirmed as 80.5%. The physical and mechanical properties of GFRP rebars at ambient temperature are shown in Table 2.

**Figure 1:**
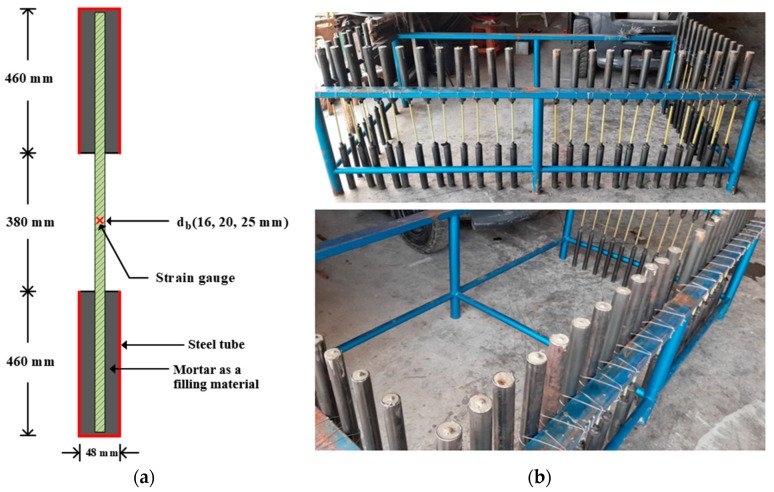
Test specimens: (**a**) details of a specimen (ASTM D7205/D7205M-06 [27]); (**b**) illustration of specimens and anchorages.

Figure 1a indicates details of a specimen that was set according to ASTM D7205/D7205M-06 [27]. All the rebars in this research were firmly covered by a steel pipe that was 460 mm long and had a diameter of 48 mm that was filled with mortar at both ends to act as interfaces between rebars and the loading head of the loading machine during the tensile tests. Installing the anchors reduced the stress concentration at the interfaces, which is observed frequently in tensile testing of GFRP rebars; hence, premature tensile failures in the GFRP rebars could be prevented to obtain the full tensile capacity of the specimens. In addition, there was a clear length of rebars between anchors of 380 mm that was heated and attached to a strain gauge to investigate the mechanical properties. The illustration of specimens and anchorages are shown in Figure 1b.

### 2.2. Heating and Cooling

**Figure 2:**
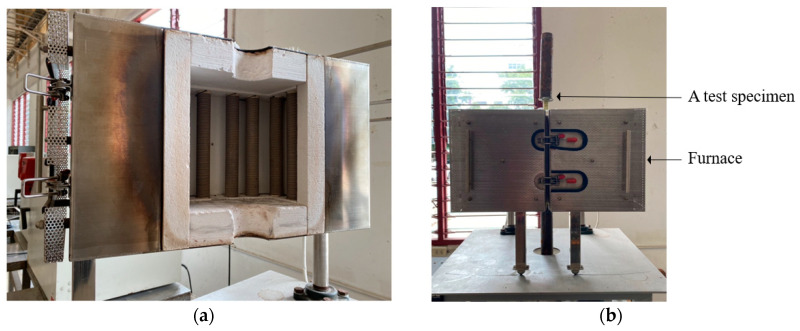
Fire testing: (**a**) internal furnace for heating test; (**b**) fire-testing setup.

Prior to the experiment, a furnace, capable of heating to 1200 °C, was modified to dimensions of 200 mm, 200 mm, and 200 mm in width, depth, and height, respectively, as shown in Figure 2a to ensure heating occurred only in the desired area. By doing so, the high temperatures were unable to influence and harm the anchors, which played a crucial role during the tensile test as interfaces between the rebars and the loading head of the loading machine.

**Figure 3:**
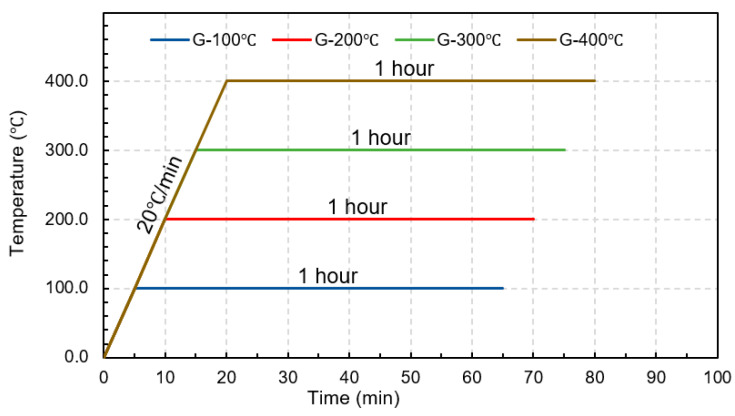
Temperature-time relationship.

During the heating process, a GFRP rebar was installed in the modified furnace, as shown in Figure 2b. Then, the specimen was heated to reach the targeted temperature using a heating rate of 20 °C/min [30], as illustrated in Figure 3. After reaching the targeted temperature, the specimen was left for 1 h to simulate a fire at the constant targeted temperature. Then, the designated cooling process was performed on the specimen.

Two techniques of cooling were investigated. The first was air cooling, with each specimen left at room temperature of about 25 °C for 24 h after the heating process. The second method used water cooling, where the burnt part of the rebar was submerged in room temperature water immediately after the heating process, and left submerged for 24 h to emulate the influences of cooling on the post-fire mechanical properties of the specimen.

### 2.3. Tensile Test

**Figure 4:**
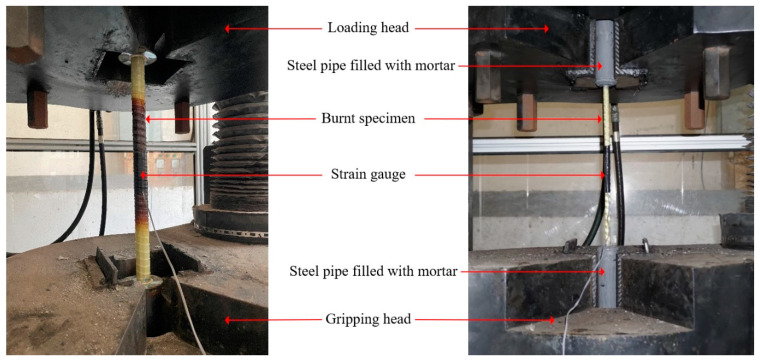
Tensile testing setup.

After allowing the specimen to cool for 24 h, the standard tensile test according to ASTM D7205/D7205M-06 [27] was conducted, as shown in Figure 4.

First, the anchorage zones of the specimen were firmly attached to the loading head of the loading machine in such a way that slipping during applying the tension force would not occur and the loading direction corresponding to the longitudinal axis of the rebar could be ensured. Next, the burnt rebar surface was polished until a satisfactory level of smoothness was achieved for strain gauge attachment. Finally, before applying the tensile load on the specimen, a strain gauge was attached at the mid-length of the clear range of the rebars for measuring the mechanical properties of the specimen after the fire.

The tensile test was performed by applying tension force to the specimen through the loading head, which was subsequently increased continuously at a loading rate of 2 mm/min [25] until failure was observed. During the loading process, the tensile stress and tensile strain were recorded to obtain a stress–strain relationship.

## 3. Results and Discussion

This section presents the results of the tensile tests of the GFRP rebars at elevated temperatures and includes the physical properties and mechanical properties. The physical properties section covers the failure modes of the FRP rebars and changes in the appearance of GFRP rebars under elevated temperatures and after the tensile tests. The mechanical properties section covers the ultimate tensile strength, ultimate strain, and elastic modulus of the GFRP rebars for the evaluated temperature and the normalized mechanical properties. It is noted that, for the purpose of clarity, a normalization approach was employed to facilitate the understanding of the post-fire mechanical properties in relation to the pre-fire properties. The normalization was defined as the ratio of the mechanical properties of the specimen after and before the fire event, was applied to present a clearer picture of the post-fire mechanical properties in comparison to the pre-fire. To illustrate, a value greater than 1.0 in the normalized mechanical properties indicates an improvement in the post-fire properties of the GFRP rebars, whereas a value lower than 1.0 signifies a decrease in the post-fire properties.

### 3.1. Mode of Failure

**Figure 5:**
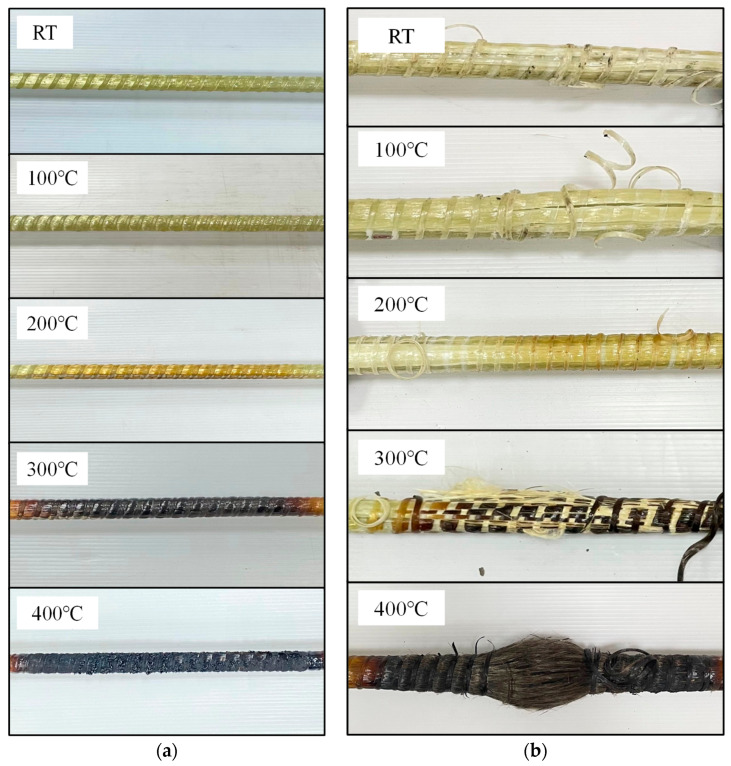
GFRP after fire exposure: (**a**) before tensile test; (**b**) failure mode after tensile test.

After removing the GFRP rebar samples from the heat treatments, samples were observed to have expanded (Figure 5a). This phenomenon may have been attributable to the high values of the resin’s coefficient of thermal expansion; this should be noted as the important point since it may contribute to the spalling of concrete under fire conditions. In addition, Figure 5a depicts the GFRP rebars exposed to varying temperatures for the same duration. The rebars exhibited almost no external color change at 100 °C. The surface yellowed when the GFRP rebars were heated to 200 °C and darkened when heated to 300 °C. By the end of their time in the oven at 400 °C, the GFRP rebars had turned an extremely dark color and the surface had turned to ashes in some parts. The hue change was attributable to the decomposition of the polymer matrix during heating. The observed hue shift was clearly correlated with the exposure temperature.

In the tensile test, regardless of the elevated temperature or cooling method, all GFRP rebars failed in a similar manner, with fiber rupture at ultimate loads. Due to surface debonding between the fibers and matrix, the failure manifested as a sudden longitudinal central fiber delamination; however, when the GFRP rebars were heated to 300 °C, a similar but more dramatic failure mode was observed, with more fibers collapsing and debonding. At 400 °C, severe delamination was replaced by fiberization. The divergent fibers of each rebar that resulted from the failure are depicted in Figure 5b. Brittle fracture was identified as the predominant mode of failure observed in all the investigated GFRP rebars in this study. This outcome is in accordance with previous studies [31], which have consistently reported the brittle fracture as the common failure mode in GFRP rebars.

### 3.2. Mechanical Properties

During the tensile test, the stress and strain of specimens were recorded and the elastic modulus was computed. The average (Avg.), standard deviation (S.D.), as well as the coefficient of variation (COV) of the experimental results are listed in Table 3.

**Figure 6:**
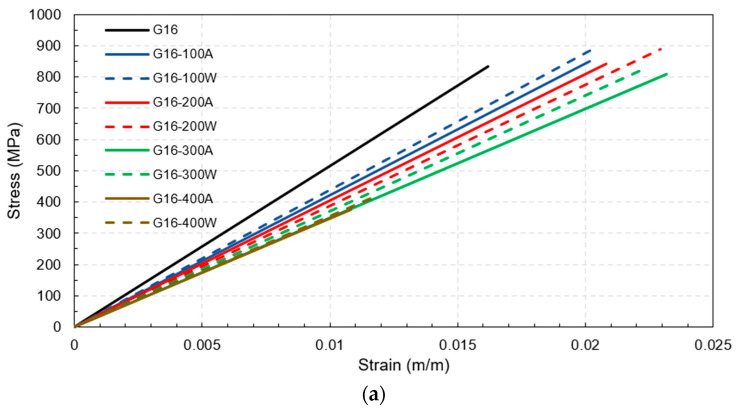

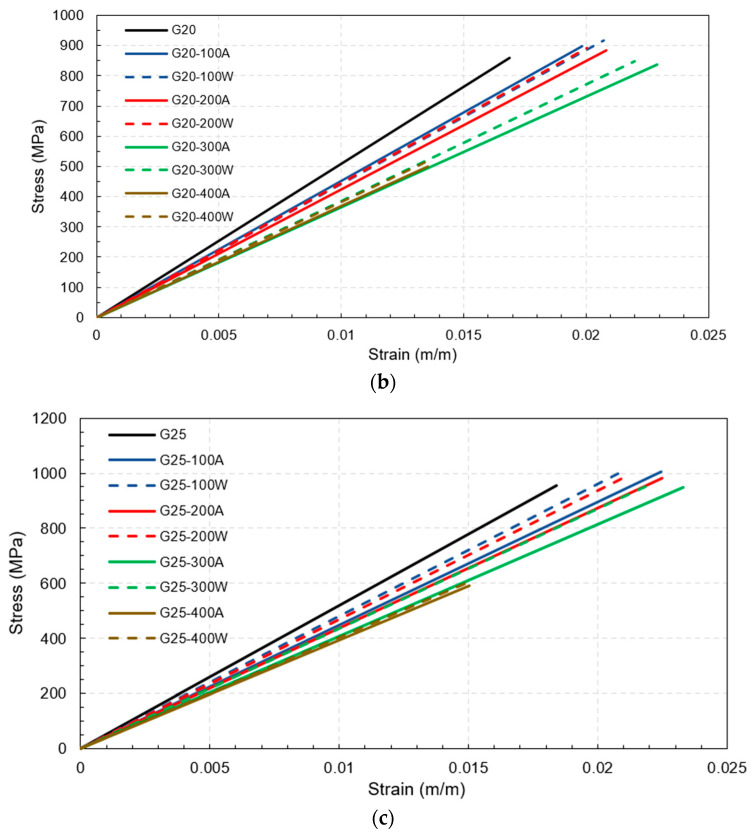
Stress–strain curves for all tested combinations: (**a**) 16 mm (**b**) 20 mm (**c**) 25 mm.

Figure 6a–c illustrate the stress–strain curves for G16, G20, and G25, respectively. Based on the test results, the stress–strain relationship of post-fire GFRP bars increased linearly from the beginning until failure. These results agreed well with Spagnuolo et al. [28], who conducted experiments to investigate the residual mechanical behavior of GFRP bars following exposure to temperature treatments ranging from 100 °C to 700 °C. Notably, their study did not specifically explore the effect of the water-cooling technique.

**Figure 7:**
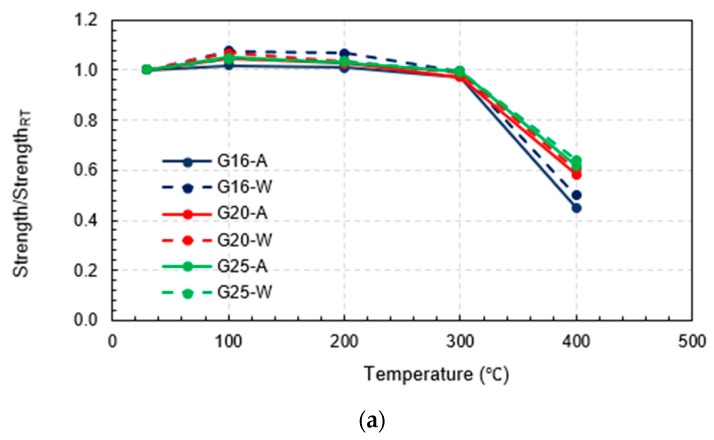

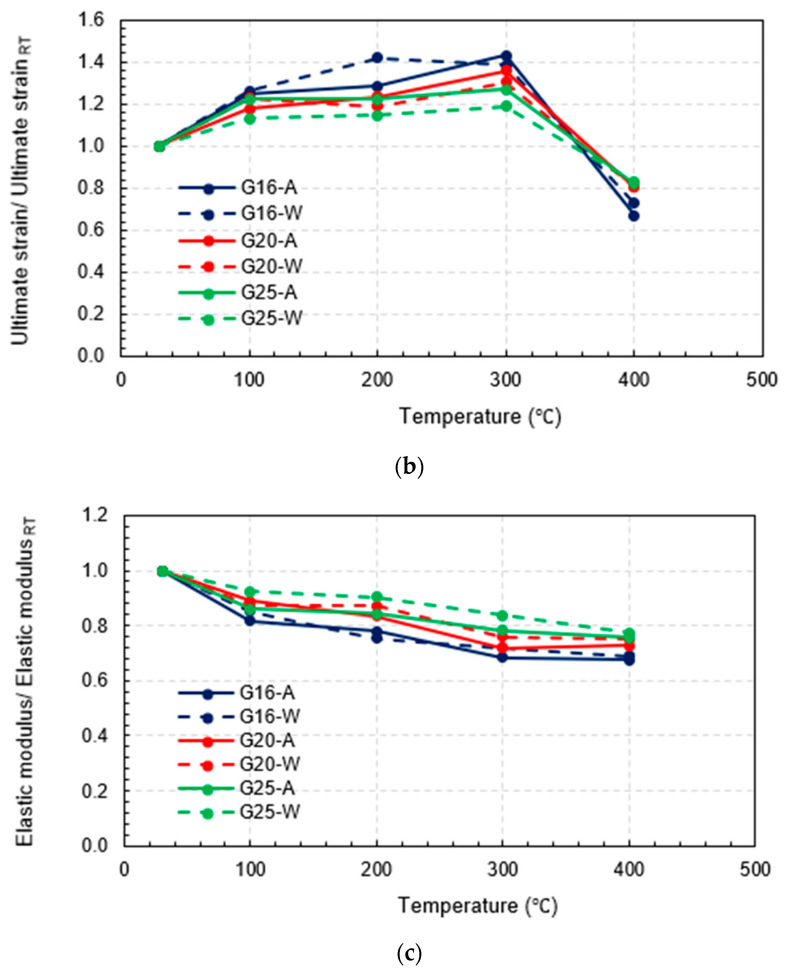
Results of all testing combinations normalized by results at room temperature: (**a**) normalized tensile strength, (**b**) normalized ultimate strain, and (**c**) normalized elastic modulus.

**Figure 8:**
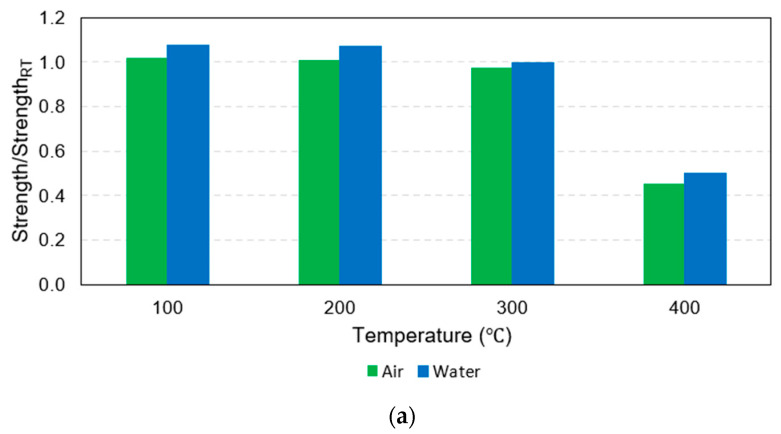

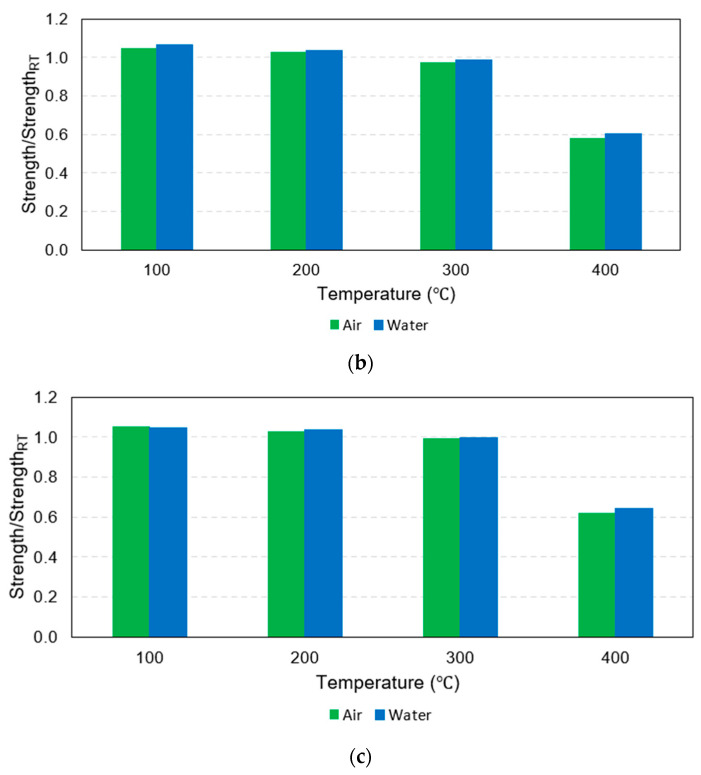
Bar charts of normalized tensile strength: (**a**) 16 mm, (**b**) 20 mm, and (**c**) 25 mm.

For the maximum temperature exposures of 100 °C, 200 °C, and 300 °C for all rebar sizes and cooling approaches, there was no significant change in the tensile strength compared to the tensile strength of identical-sized rebars before the fire because the maximum temperature exposures did not exceed the critical temperature of between 300 °C and 400 °C; hence, the mechanical properties after being cooled and tested had recovered. Notably, even though there were slight increments in the tensile strength after exposure to this range of elevated temperatures, these increments might have been inconsistent with the real behavior of the rebars in use because the increases may have been the consequence of better curing of the resin when there was no applied load on the fiber direction of the rebars during the fire, as demonstrated in this study [28]. On the other hand, in cases where the targeted temperature reached 400 °C, the rebars experienced severe damage when they reached the critical temperature range of 300 °C to 400 °C. This resulted in thermal degradation of the polymer and loss of load transfer among the fibers. Consequently, the mechanical properties became non-reversible. This observation is consistent with the findings of a previous study [32] which investigated the tensile, shear, and flexural properties of sand-coated GFRP bars under low temperatures (−100 °C to 0 °C) and elevated temperatures (23 °C to 315 °C). The purpose of that study was to gain insights into the thermal stability of the mechanical properties. Thus, the tensile strengths of G16, G20, and G25 decreased substantially by about 55%, 41%, and 38%, respectively, regardless of the cooling approach, as can be clearly observed from the plot of normalized tensile strength in Figure 7a. Additionally, these numbers implied that the larger the rebar in the range between 16 mm and 25 mm, the smaller the percentage of tensile strength reduction when exposed to 400 °C. It can be seen from Figure 8 that the residual tensile strengths of all rebars after cooling using water were slightly greater than those that were gradually cooled using air.

**Figure 9:**
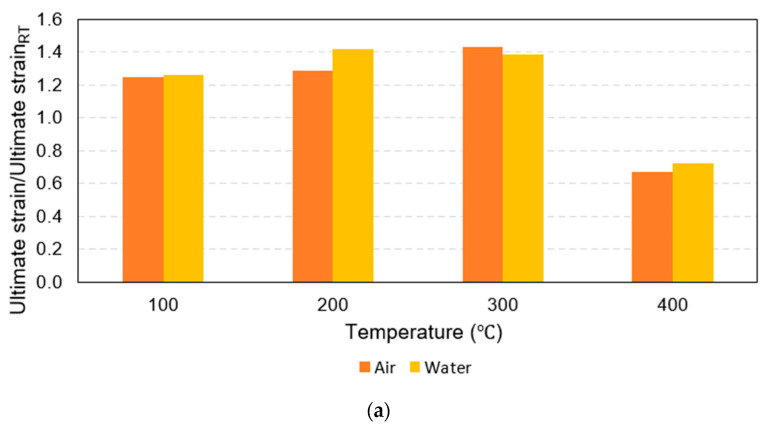

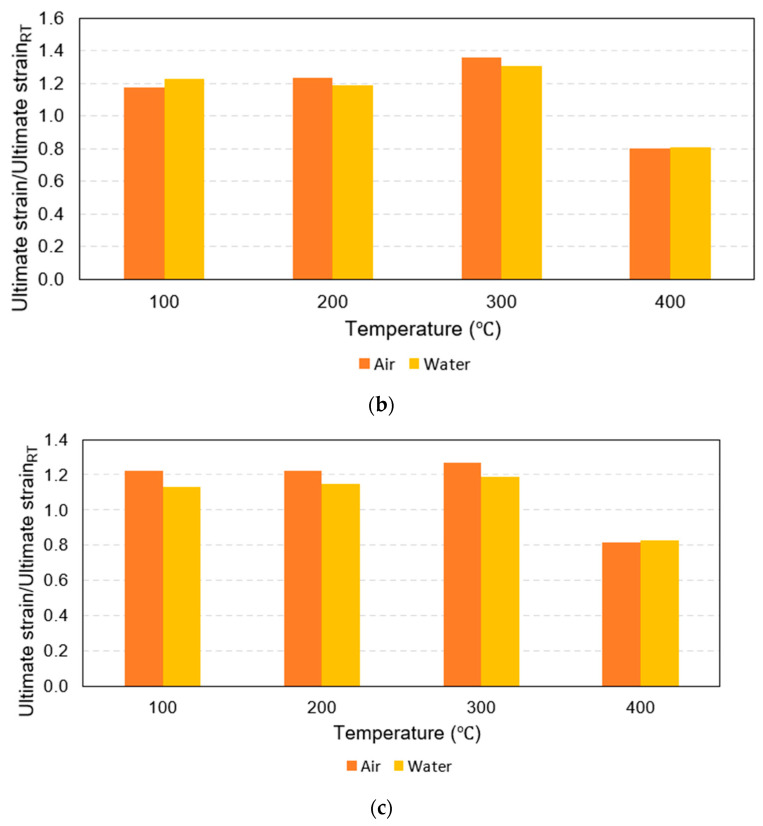
Bar charts of normalized ultimate strain: (**a**) 16 mm, (**b**) 20 mm, and (**c**) 25 mm.

Considering the ultimate strain, it was clear that all the rebars that were exposed to 100 °C, 200 °C, and 300 °C deformed more than the same-sized rebars before the fire. In other words, rebar ductility increased due to temperature exposure between 100 °C and 300 °C. On the other hand, for maximum temperature exposures to 400 °C, the plot of the normalized ultimate strain in Figure 7b indicates that the ultimate strain of G16, G20, and G25 decreased significantly compared to the pre-fire identical size rebars by about 33%, 20%, and 18%, respectively. Similar to the effects due to the rebar size on the post-fire tensile strength reduction, after exposure to 400 °C, the larger rebars within the range 16–25 mm tended to be more ductile than the smaller rebars in the same range and the percentage of the ductility reduction was relatively lower. In addition, Figure 9 shows that the ultimate strain levels of all rebars after exposure to 400 °C followed by water cooling were more than for the air-cooled rebars, while the ultimate strain levels of the 25 mm rebars after being heated to 100 °C, 200 °C, and 300 °C before being air cooled by air were slightly more than those that were water cooled. However, there was no clear trend observed in the other specimens.

**Figure 10:**
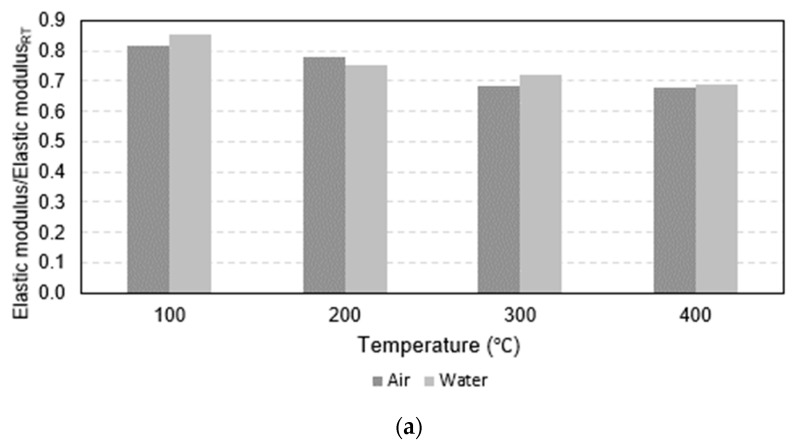

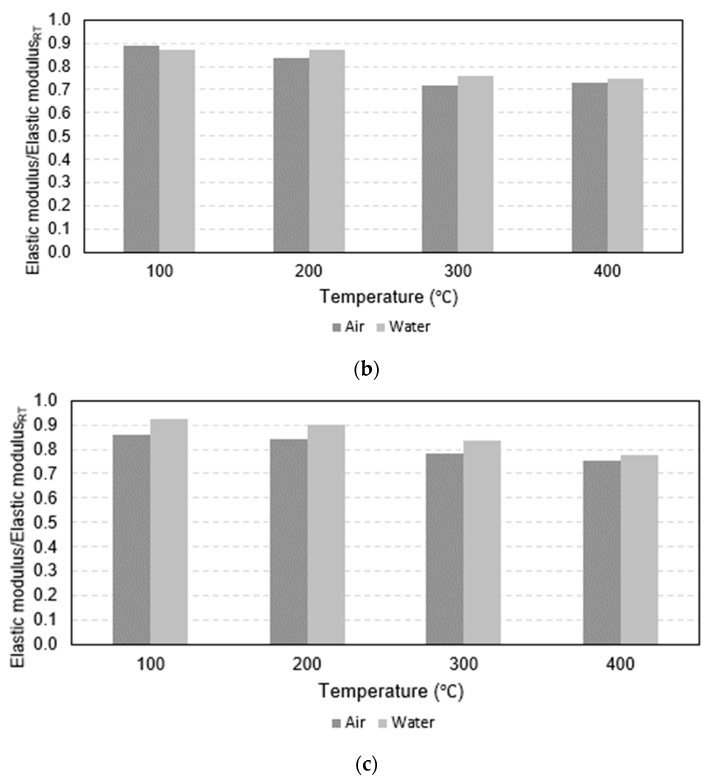
Bar charts of normalized elastic modulus: (**a**) 16 mm, (**b**) 20 mm, and (**c**) 25 mm.

The normalized elastic modulus in Figure 7c demonstrated a clear pattern that as maximum temperature increased, the modulus of elasticity decreased. In addition, some specimens (16 mm and 20 mm rebars heated to 300 °C and 400 °C before being cooled by water as well as all 25 mm water-cooled rebars) had a greater modulus of elasticity compared to those that were air cooled. However, Figure 10 shows that these relationships did not hold true for the 16 mm and 20 mm rebars heated to maximum temperatures of 200 °C and 100 °C, respectively.

Notably, in the case of traditional steel bars, previous studies such as [33,34] have collected data on the post-fire mechanical properties. It has been observed that the cooling technique exerts a significant influence on these properties. For instance, water-cooled steel bars tend to experience reductions in strength recovery and ductility after exposure to temperatures exceeding 600 °C [33]. However, it is important to clarify that despite a substantial decrease in ultimate strain in water-cooled steel bars by nearly 50%, they still exhibit a notable increase in post-fire strength compared to air-cooled steel bars [34]. However, even though the same results were observed in all the GFRP rebars exposed to more than 300 °C, the differences were far lower than those investigated for steel bars. Thus, it was not reliable to compare the higher strength of the water-cooled GFRP rebars to the air-cooled ones in post-fire evaluation.

### 3.3. Variation Model of Post-Fire Mechanical Properties

**Figure 11:**
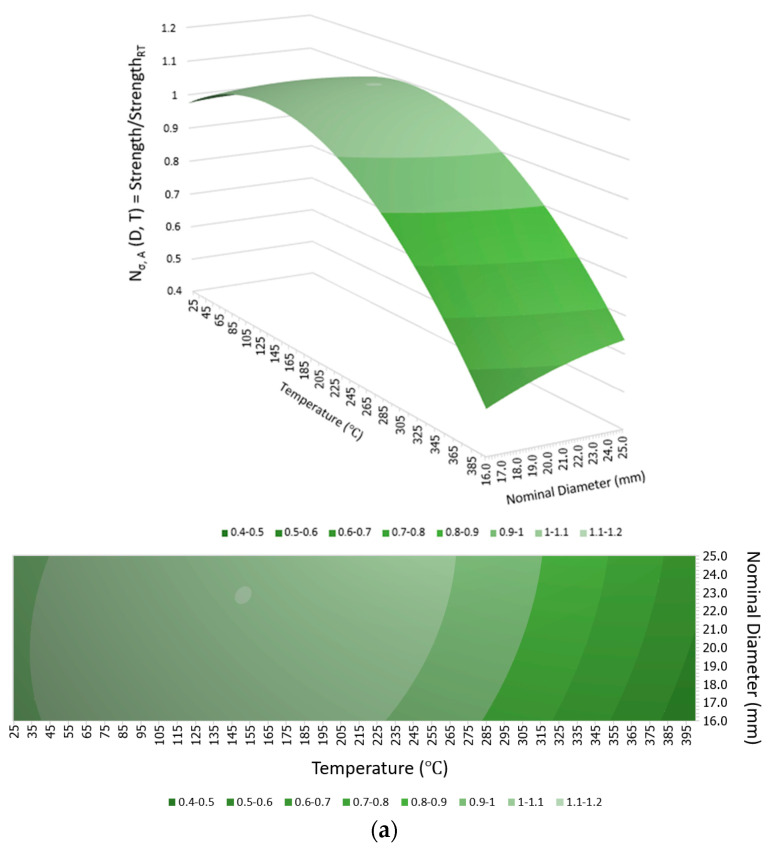

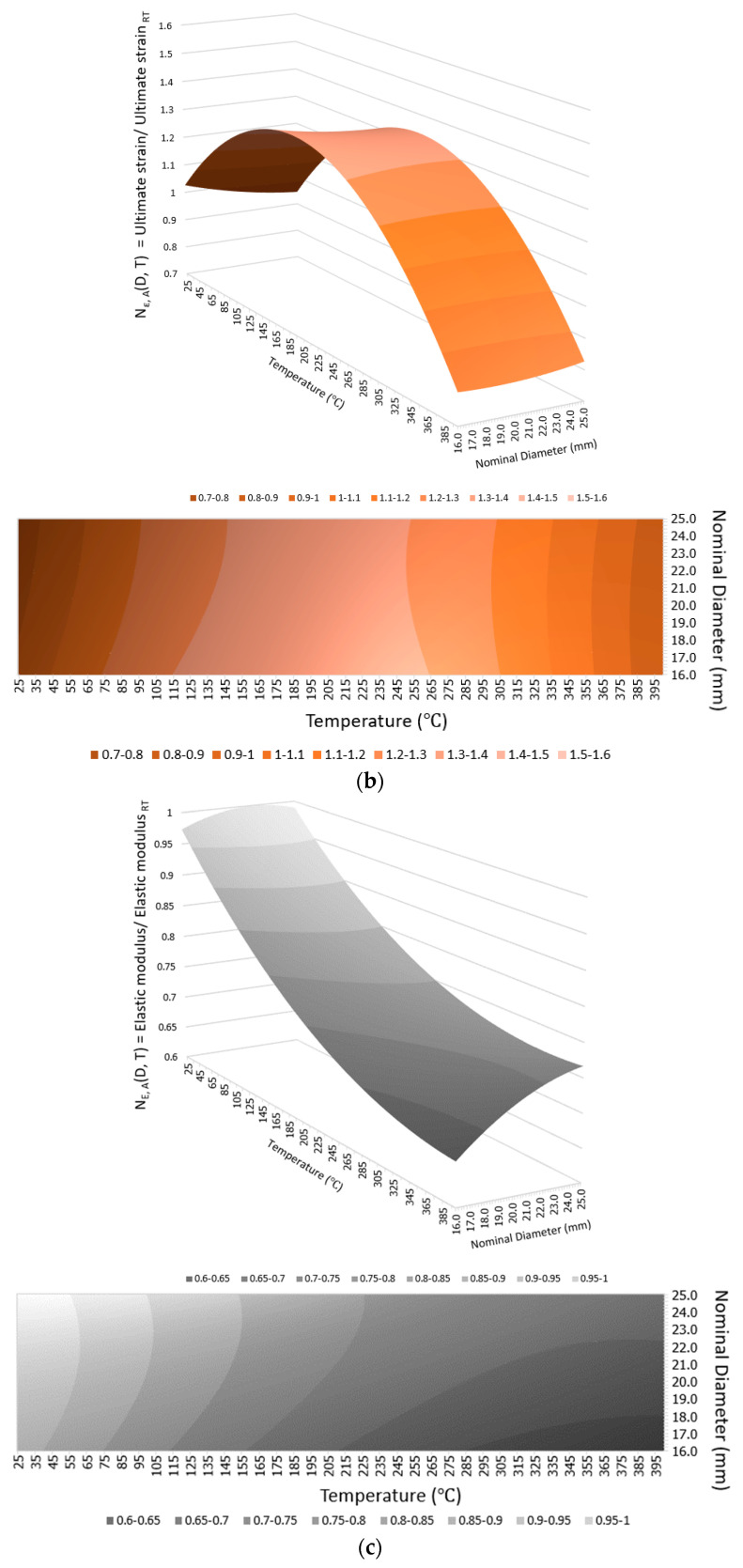
Models of mechanical properties of various nominal diameter GFRP bars after exposure to different maximum fire temperatures followed by air cooling: (**a**) strength variation, (**b**) ultimate strain variation, and (**c**) elastic modulus variation.

**Figure 12:**
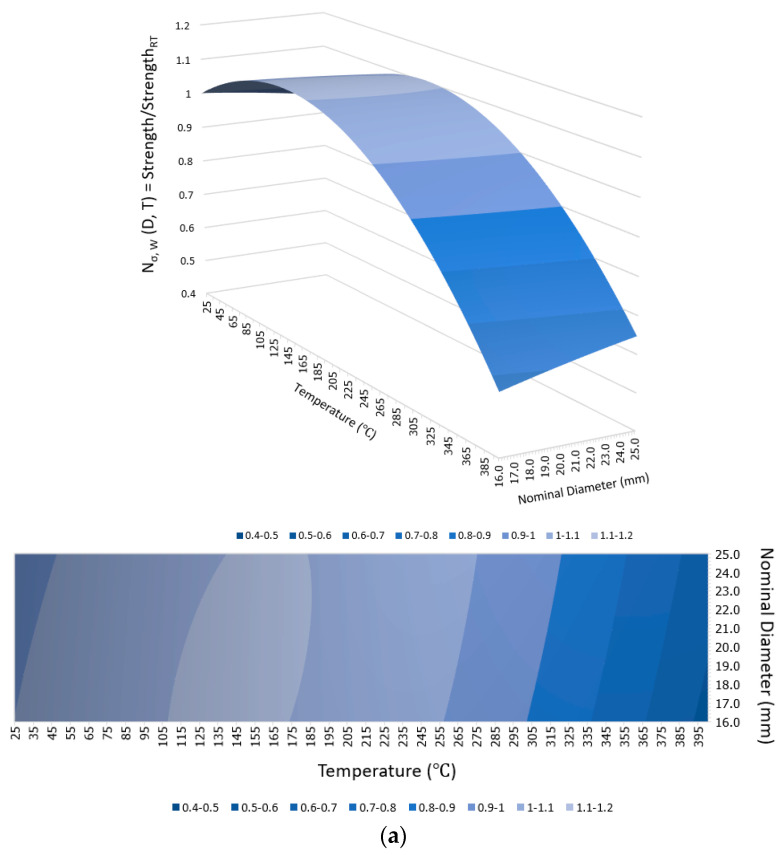

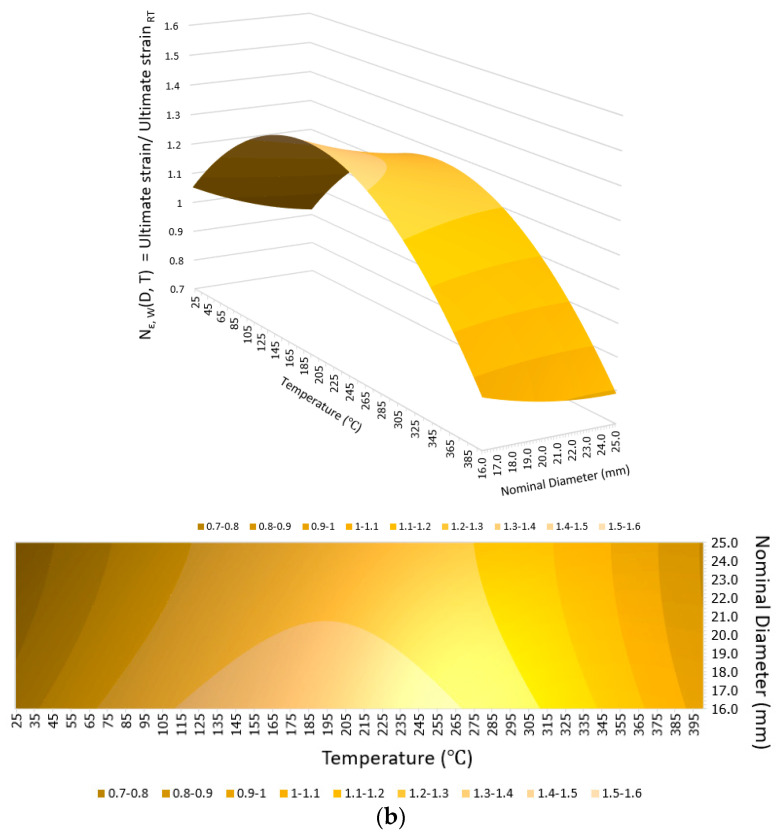

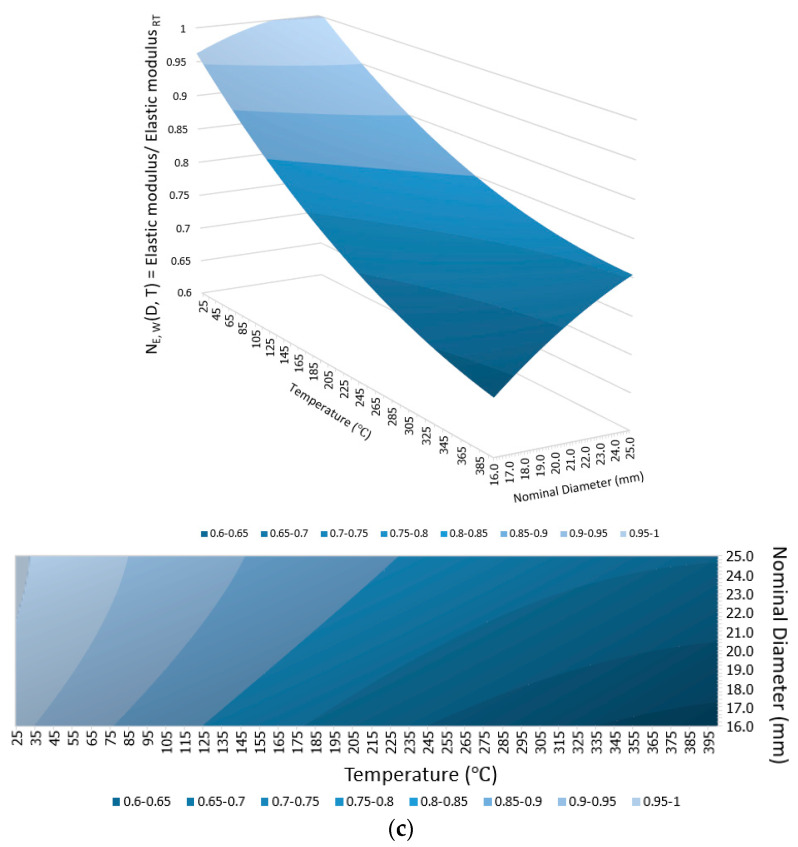
Models of mechanical properties of various nominal diameters of GFRP bars after exposure to different maximum fire temperatures followed by water cooling: (**a**) strength variation, (**b**) ultimate strain variation, and (**c**) elastic modulus variation.

The mechanical properties of the GFRP rebars were normalized using the pre-fire properties to provide an indication of post-fire mechanical properties variation. These normalized properties, namely N_i,j_ where i denotes strength (σ), ultimate strain (ε), and elastic modulus (E) while j denotes the implemented cooling method (A and W for the air and water cooling method, respectively), were plotted against the maximum fire temperature exposures as well as the nominal diameters in Figure 11 and Figure 12, respectively, for air and water cooling, respectively, to show the more apparent course of the correlation between these two parameters and the dependent variables when implementing different cooling methods. After that, the variation trend was fitted with a quadratic response surface model, as shown in Equation (1):

N_i,j_(D,T) = β_1_D^2^ + β_2_T^2^ + β_3_DT + β_4_D + β_5_T + β_6_ (1)

where D and T are the nominal diameter (mm) and the maximum fire temperature exposure (°C), respectively. β_1_–β_6_ are the fitted coefficients of the above quadratic response surface, as listed in Table 4.

## 4. Conclusions

This study investigated the post-fire mechanical properties of GFRP rebars, based on rebars with nominal diameters of 16 mm, 20 mm, and 25 mm, maximum temperature exposures between 100 °C and 400 °C, and air-cooling techniques and water-cooling methods. In total, 135 specimens were heated to reach the targeted maximum temperature, followed by continual exposure at that temperature for 1 h before being cooled using the designated cooling approach for 24 h. Finally, tensile tests were conducted on the rebars to obtain the post-fire mechanical properties. The following conclusions were drawn:

The modulus of elasticity of the GFRP rebars decreased as the maximum fire temperature increased compared to the pre-fire modulus of elasticity of the identical-sized rebar.

- Tensile strength reductions were observed in all rebars heated to 400 °C; ductility increased up to 300 °C before decreasing significantly at 400 °C.
- The ductility of GFRP rebars increased due to temperature exposures between 100 °C and 300 °C. However, the ultimate strain decreased significantly after the temperature increased to 400 °C. After exposure to 400 °C, the percentage of tensile strength and ductility reductions decreased as the size of the rebars increased within the range 16–25 mm.
- The post-fire tensile strength of the GFRP rebars that were water cooled was slightly greater than that of the same-sized rebars that were air cooled except for the case of 25 mm bars that were subjected to the maximum temperature of 100 °C. The tensile strength, ultimate strain, and modulus of elasticity of all rebars cooled using water after exposure to temperatures exceeding the critical temperature of between 300 °C and 400 °C, i.e., the case of maximum temperature exposure reach 400 °C in this study were slightly higher than those that had been gradually cooled at ambient temperature. It has been observed that the effects of cooling methods, as observed in this study, are minimal and inconsequential. Furthermore, slight fluctuations in the results were noted.
- The correlations between maximum temperature exposure, rebar nominal diameter, cooling method, and the post-fire mechanical properties were plotted, and variation models were developed by fitting the correlations with the quadratic response surface model. The adoption of this model was based on its capability to provide a reliable reference for design purposes, given its favorable shape.

However, considerations of the physical characteristics, mechanical behavior, and changes in the conditions of reinforcing materials are just some of the important factors that contribute to improving and selecting suitable options for different types of structures. When designing reinforced concrete building elements, it is crucial to consider also the concrete design and to have sufficient concrete cover to ensure proper usage. Incorporating the findings from the study on the behavior of these reinforcing materials into the design process can result in the establishment of appropriate design boundaries. Furthermore, it can lead to a reduction in the consumption of concrete materials by minimizing the concrete cover.

## Figures and Tables

**Table 1:** Test specimens, where RT = room temperature.

Test Code	Diameter (mm)	Temperature (°C)	Cooling Method
G16	16	RT	-
G20	20	RT	-
G25	25	RT	-
G16-100A	16	100	Air
G16-200A	16	200	Air
G16-300A	16	300	Air
G16-400A	16	400	Air
G16-100W	16	100	Water
G16-200W	16	200	Water
G16-300W	16	300	Water
G16-400W	16	400	Water
G20-100A	20	100	Air
G20-200A	20	200	Air
G20-300A	20	300	Air
G20-400A	20	400	Air
G20-100W	20	100	Water
G20-200W	20	200	Water
G20-300W	20	300	Water
G20-400W	20	400	Water
G25-100A	25	100	Air
G25-200A	25	200	Air
G25-300A	25	300	Air
G25-400A	25	400	Air
G25-100W	25	100	Water
G25-200W	25	200	Water
G25-300W	25	300	Water
G25-400W	25	400	Water

**Table 2:** Physical and mechanical properties of GFRP rebars.

Property	Glass Content	Tensile Strength	Tensile Modulus	Ultimate Strain
Value	80.5% (by mass)	930 MPa	50.2 GPa	0.0185

**Table 3:** Experimental results.

Specimen	Tensile Stress (MPa)			Strain (m/m)			Elastic Modulus		
	Avg. (MPa)	S.D. (MPa)	COV	Avg. (m/m)	S.D. (m/m)	COV	Avg. (GPa)	S.D. (GPa)	COV
G16	833.0	9.9	1.2	0.0162	0.00014	0.9	51.5	0.1	0.1
G20	859.4	10.9	1.3	0.0169	0.00016	0.9	50.9	0.2	0.3
G25	955.1	14.1	1.5	0.0184	0.00066	3.6	52.0	2.6	5.1
G16-100A	849.0	11.3	1.3	0.0218	0.00093	4.6	42.1	1.4	3.3
G16-200A	842.0	2.8	0.3	0.0201	0.00030	1.5	40.1	1.4	3.5
G16-300A	809.5	17.7	2.2	0.0232	0.00206	8.9	35.3	3.7	10.5
G16-400A	375.8	17.5	4.7	0.0108	0.00016	1.5	34.7	2.1	6.2
G16-100W	895.4	58.5	6.5	0.0204	0.00075	3.7	43.9	1.2	2.9
G16-200W	899.5	12.0	1.4	0.0230	0.00019	0.8	38.8	0.8	2.2
G16-300W	829.4	15.6	1.9	0.0224	0.00007	0.3	37.0	0.6	1.6
G16-400W	415.7	9.9	2.4	0.0117	0.00083	7.1	35.5	1.7	4.7
G20-100A	898.5	3.7	0.4	0.0198	0.00024	1.2	45.3	0.7	1.6
G20-200A	883.5	7.9	0.9	0.0208	0.00040	1.9	42.4	0.4	1.0
G20-300A	835.7	5.7	0.7	0.0229	0.00140	6.1	36.5	2.0	5.4
G20-400A	500.9	5.4	1.1	0.0135	0.00048	3.5	37.0	0.9	2.4
G20-100W	917.3	10.6	1.2	0.0207	0.00094	4.5	44.3	1.5	3.4
G20-200W	891.3	9.4	1.1	0.0201	0.00003	0.1	44.4	0.5	1.2
G20-300W	849.0	8.3	1.0	0.0212	0.00073	3.3	38.6	0.9	2.3
G20-400W	518.3	9.1	1.8	0.0136	0.00017	1.3	38.2	1.2	3.0
G25-100A	1004.1	11.6	1.2	0.0225	0.00055	2.5	44.8	1.6	3.6
G25-200A	982.8	4.6	0.5	0.0225	0.00111	4.9	43.8	2.4	5.4
G25-300A	947.1	7.5	0.8	0.0233	0.00091	3.9	40.7	1.3	3.1
G25-400A	590.7	9.0	1.5	0.0150	0.00005	0.3	39.3	0.5	1.2
G25-100W	997.6	36.9	3.7	0.0207	0.00056	2.7	48.0	0.5	1.0
G25-200W	989.3	7.5	0.8	0.0211	0.00062	2.9	46.9	1.0	2.2
G25-300W	950.3	22.3	2.3	0.0218	0.00035	1.6	43.5	1.7	1.6
G25-400W	611.3	9.3	1.5	0.0152	0.000001	0.01	40.3	0.6	1.5

**Table 4:** Fitted coefficients of Equation (1), where R^2^ = coefficient of determination.

Normalized Mechanical Property	β_1_	β_2_	β_3_	β_4_	β_5_	β_6_	R^2^
N_σ,A_(D,T)	−7.03 × 10^−4^	−7.78 × 10^−6^	3.73 × 10^−5^	2.65 × 10^−2^	1.50 × 10^−3^	6.84 × 10^−1^	0.93
N_σ,W_(D,T)	−1.68 × 10^−4^	−8.04 × 10^−6^	3.72 × 10^−5^	6.98 × 10^−4^	1.67 × 10^−3^	9.81 × 10^−1^	0.94
N_ε,A_(D,T)	5.75 × 10^−4^	−1.28 × 10^−5^	3.03 × 10^−5^	−3.34 × 10^−2^	4.36 × 10^−3^	1.30	0.79
N_ε,W_(D,T)	8.78 × 10^−4^	−1.17 × 10^−5^	2.07 × 10^−5^	−5.14 × 10^−2^	4.13 × 10^−3^	1.54	0.84
N_E,A_(D,T)	−8.41 × 10^−4^	2.00 × 10^−6^	2.52 × 10^−5^	3.57 × 10^−2^	−2.08 × 10^−3^	6.58 × 10^−1^	0.95
N_E,W_(D,T)	−4.99 × 10^−4^	1.33 × 10^−6^	2.39 × 10^−5^	2.50 × 10^−2^	−1.71 × 10^−3^	7.23 × 10^−1^	0.94

## Data Availability

The data presented in this study are available on request from the corresponding author.
